# Natural Compound Oridonin Inhibits Endotoxin-Induced Inflammatory Response of Activated Hepatic Stellate Cells

**DOI:** 10.1155/2018/6137420

**Published:** 2018-12-30

**Authors:** Claire B. Cummins, Xiaofu Wang, Christian Sommerhalder, Frederick J. Bohanon, Omar Nunez Lopez, Hong-Yan Tie, Victoria G. Rontoyanni, Jia Zhou, Ravi S. Radhakrishnan

**Affiliations:** ^1^Department of Surgery, University of Texas Medical Branch, Galveston, Texas, 77555, USA; ^2^Department of Anatomy, College of Basic Medicine, Zhengzhou University, Zhengzhou 450066, China; ^3^Department of Surgery, Shriners Hospitals for Children, Galveston, Texas, 77555, USA; ^4^Department of Pharmacology and Toxicology, University of Texas Medical Branch, Galveston, Texas, 77555, USA

## Abstract

Hepatic stellate cells (HSCs) play an important role in hepatic fibrogenesis and inflammatory modulation. Endotoxin is dramatically increased in portal venous blood after serious injury and can contribute to liver damage. However, the mechanism underlying endotoxin's effects on HSCs remains largely unknown. Oridonin is a bioactive diterpenoid isolated from* Rabdosia rubescens* that exhibits anti-inflammatory properties in different tissues. In the present study, we determined the effects of oridonin on endotoxin-induced inflammatory response and signaling pathways* in vitro.* The production of proinflammatory cytokines in activated human HSCs line LX-2 was measured by ELISA and Western blots. Immunofluorescence and nuclear fractionation assay were used to determine NF-*κ*B activity. Oridonin treatment significantly inhibited LPS-induced proinflammatory cytokines IL-1*β*, IL-6, and MCP-1 production as well as cell adhesion molecules ICAM-1 and VCAM-1. Additionally, oridonin blocked LPS-induced NF-*κ*B p65 nuclear translocation and DNA binding activity. Oridonin prevented LPS-stimulated NF-*κ*B regulator IKK*α*/*β* and I*κ*B*α* phosphorylation and I*κ*B*α* degradation. Combined treatment of oridonin and an Hsp70 substrate binding inhibitor synergistically suppressed LPS-stimulated proinflammatory cytokines and NF-*κ*B pathway activation. Therefore, oridonin inhibits LPS-stimulated proinflammatory mediators through IKK/I*κ*B*α*/NF-*κ*B pathway. Oridonin could be a promising agent for a hepatic anti-inflammatory.

## 1. Introduction

*Rabdosia rubescens*, a Chinese medicinal herb, contains the active diterpenoid compound oridonin. For hundreds of years,* Rabdosia rubescens* has been used in Chinese traditional medicine as an antitumor, antimicrobial, anti-inflammatory, and antioxidant agent. It has been used to treat a variety of ailments including stomach aches, pharyngitis, cough and sport-related injuries, as well as multiple cancer types, such as esophageal, breast, liver, and prostate [1, 2]. Oridonin was identified in the late 1960s as the active compound in* Rabdosia rubescens* and was able to be synthesized in early 1970s. Today, it remains one of the most important compounds isolated from traditional Chinese herbs. Oridonin has been shown to have multiple biological activities. Oridonin has been shown to interfere with several pathways involved in cell proliferation, cell cycle arrest, and apoptosis, which contribute to its chemopreventative and antitumor effects. [3, 4]. Recently, several research papers have demonstrated that oridonin also has immunomodulatory effects on the immune system and proinflammatory mediators. For example, in murine primary splenocytes, oridonin significantly inhibited both IL-2 (Th1) and IL-10 (Th2) cytokine production at the same time, suggesting that this terpenoid compound has anti-inflammatory potential through the inhibition of T-cell immune responses [5]. The NF-*κ*B pathway has been well established as playing a prominent role in inflammatory processes. Oridonin was found to inhibit NF-*κ*B activity in different cell types [6, 7]. Importantly, Zhou and colleagues [8] reported that oridonin showed significant antileukemic and organ protective effects without obvious adverse reaction. Their results revealed that oridonin treatment markedly reduced disseminated disease and prevented the destruction of tissue architecture caused by leukemia in both the liver and the spleen, indicating that oridonin exerts a protective effect on the liver. Multiple studies have further demonstrated the ability of oridonin to block inflammatory signaling in other tissue such as renal cortex tissue, colonic tissue, hippocampal tissue, BxPC-3 pancreatic cancer cells, and U937 human histocytic lymphoma cells [9–13]. However, the effect of oridonin on liver inflammation remains largely unknown, and our study is the first to examine its role in hepatic stellate cells (HSCs).

Quiescent HSCs are nonproliferative and are typically localized within the space of Disse and function as the principal storage site of retinoids in the liver. Upon activation, HSCs proliferate, and are primarily characterized as the main effector cells for liver fibrosis, due to their capacity to transdifferentiate into collagen-producing myofibroblasts. More recent studies have elucidated the fundamental role of HSCs not only in hepatic fibrogenesis but also in liver immunology. HSCs have been reported to respond to many immunological triggers via toll-like receptors (TLR) (e.g., TLR4, TLR9) as well as transduction signals through pathways and mediators traditionally found in immune cells, such as NF-*κ*B and the Hedgehog (Hh) pathways or inflammasome activation [14–16]. Activated HSCs are identified as a versatile source of many soluble immunologically active factors including cytokines and chemokines and may act as antigen presenting cells. After liver injury, HSCs are important sensors of altered tissue integrity and initiators of innate immune cell activation [17]. Previously, our group demonstrated the antifibrogenic effects of oridonin and its analogs in activated HSC lines via inhibition of cell proliferation and suppression of extracellular matrix expression, such as collagen type I and fibronectin [18–20]. In the present study, we determined the immunomodulatory effects of oridonin with the activated human HSC line LX-2. Our main objective was to examine the effects and molecular mechanism of oridonin on proinflammatory mediators and adhesion molecules stimulated by lipopolysaccharide (LPS), which is dramatically increased in portal venous blood after serious injuries. We hypothesized that oridonin would inhibit the LPS-induced inflammatory response in HSCs.

## 2. Materials and Methods

### 2.1. Reagents

All cell culture mediums and trypsin were purchased from Life Technology Corp. (Carlsbad, CA). We purchased oridonin from Sigma-Aldrich (St. Louis, MO). Hsp70 substrate binding inhibitor PES-CI was from EMD Millipore (Now is Millipore Sigma). The following primary antibodies were used: rabbit anti- NF-*κ*B p65 (#8242), rabbit anti- I*κ*B*α* (#4814), rabbit anti- IKK-*β* (#8943), rabbit anti-IKK*α* (#11930), rabbit anti-phospho I*κ*B*α* Ser32 (Cat#2859), and rabbit anti-Glyceraldehyde 3-phosphate dehydrogenase (GADPH) (#5174) were from Cell Signaling Technology Inc., Danvers, MA); mouse anti-IL-1*β* (sc-7884), mouse anti-ICAM-1 (sc-8439), VCAM-1(sc-8304), and goat anti-Lamin A (sc-71481) were from Santa Cruz Biotechnology(Santa Cruz, CA); mouse anti-Topo II *β* (#3611492) was from BD Bioscience (San Jose, CA); rabbit anti-phospho IKK*α*/*β* (ab195907) was abcam (Cambridge, MA).

### 2.2. Cell Culture

As described in previous publications, the human immortalized HSC line LX-2 immortalized HSC line was a gift from Dr. Scott Friedman (Mount Sinai Medical Center, New York) and cultured at 37°C with 5% CO_2_ in Dulbecco's modified Eagle's medium (DMEM) with a high glucose concentration (4.5 g/L) supplemented with 5% fetal bovine serum (FBS) and 1% penicillin/streptomycin. [20, 21]. Within 6 weeks of culture from liquid nitrogen, all experiments were performed.

### 2.3. Nuclear Protein Extraction and NF-*κ*B p65 DNA-Binding Activity

Nuclear protein was isolated with NE-PER nuclear and cytoplasmic extraction reagent (Pierce Biotechnology; Rockford, IL) according to the instructions of the manufacturer. The Trans-AM NF-*κ*B p65 Transcription Factor kit was purchased from Active Motif North America (Carlsbad, CA) and the manufacturer's instructions were followed in order to quantify the DNA binding activity of NF-*κ*B. The wild-type consensus oligonucleotide was used as a competitor for NF-*κ*B binding, as according to the instructions. This competition assay confirms that the protein subunits binding to the plate are specific for the NF-*κ*B consensus binding sequence and is used as a control for each cell type studied. The samples were analyzed in duplicate and repeated at least three times.

### 2.4. Western Immunoblotting

As described in previous publications [20, 21], RIPA buffer (Thermo Fischer Scientific, Inc., Waltham, MA) with 1% Halt protease inhibitor cocktail and 1% Halt phosphatase inhibitor cocktails (Thermo Fischer Scientific, Inc., Waltham, MA) was used to prepare whole cell extracts. NE-PER nuclear and cytoplasmic extraction reagent (Pierce Biotechnology) were used to prepare nuclear extracts. The Bradford method was used to measure and quantify the protein concentration. 10-30 g of protein were fractionated by sodium dodecyl sulfate-polyacrylamide gel electrophoresis (SDS-PAGE) (Life Technologies Corporation, Grand Island, NY) under denaturing conditions and then electro-transferred to a polyvinylidene fluoride (PVDF) membrane. After being blocked with Blocking buffer (LI-COR, Inc., Lincoln, NE) the membrane was probed with the indicated primary antibody (Ab) diluted with blocking buffer. Membranes were washed three times with Phosphate buffered saline with 0.1% Tween 20 (PBST) and incubated 1 h with IRDye 680-conjugated anti-mouse or IRDye 800-conjugated anti-rabbit Ab (LI-COR, Inc., Lincoln, NE). Finally, the membranes were washed three times with PBST, and signals were scanned and visualized by Odyssey Infrared Imaging System (LI-COR, Inc., Lincoln, NE). Densitometric analysis was performed on the proteins of interest and normalized to GAPDH by LI-COR Image Studio software (LICOR, Inc., Lincoln, NE).

### 2.5. Immunoflourescence Staining

After indicated treatments, the cells were fixed with 4% paraformaldehyde in PBS for 20 min, and permeabilized with 0.3% Triton-100 in PBS, followed by incubation in blocking buffer (5% goat serum, 0.1% triton X-100 in PBS) and incubated with primary antibodies in incubation buffer (0.1% triton X-100 and 1% goat serum in PBS) overnight at 4°C. After three times washing with PBS, cells were stained with Alexa Fluor 488-conjugated Goat anti-rabbit IgG (Invitrogen) in incubation buffer for 1 h. After removing secondary antibody, the cells were fixed and counterstained with DAPI. The cells were visualized by Nikon fluorescence LSM510 confocal microscope at 20X magnification.

### 2.6. Enzyme-Linked Immunosorbent Assay (ELISA)

Twenty-four hours after cell treatment, the culture supernatant was corrected for subsequent measurement of cytokines secretion. The supernatants were measured using commercially available human IL-6 ELISA kit (#KHC0061) and human MCP-1 ELISA kit (#KHC1011) from Life Technologies (Frederick, MD) according to the instructions of the manufacturer. The total amount IL-6 and MCP-1 in the cell medium was normalized to the total amount of protein in the viable cell pellets. The samples were analyzed in duplicate and repeated at least three times.

### 2.7. Statistical Analysis

Statistical analysis was performed with GraphPad Prism 7.0 from GraphPad Software Inc. (La Jolla, CA, USA) as previously described [21].Where indicated, one-way ANOVA with Sidak's multiple comparisons test were used. All figures are presented as mean ± SEM, with the following significance denotation *∗*: P<0.05, *∗∗*: P<0.01, and *∗∗∗*: P<0.001.

## 3. Results

### 3.1. Oridonin Inhibits LPS-Induced Inflammatory Cytokines and Cell Adhesion Molecules

**Figure 1:**
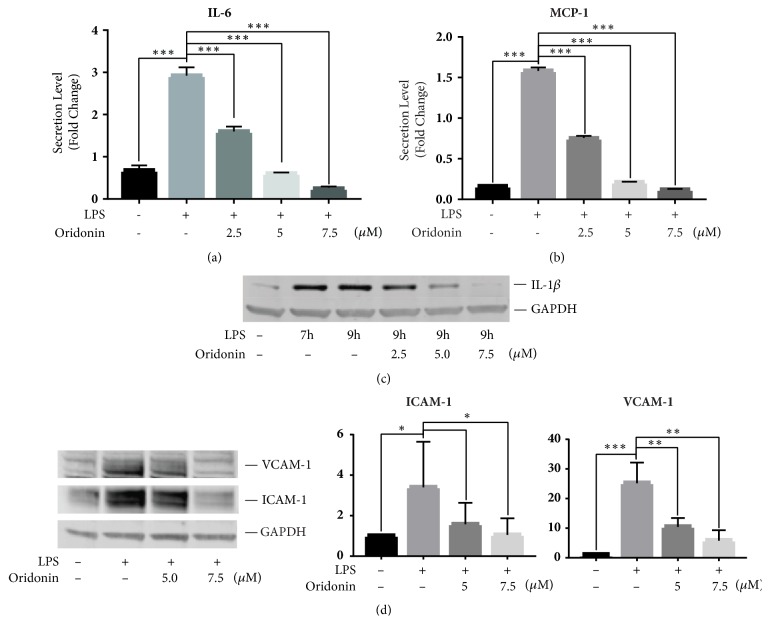
*Oridonin inhibits LPS-induced pro-inflammatory cytokines and cell adhesion molecules*. LX-2 cells were pretreated with oridonin (2.5, 5, or 7.5 *μ*M) for 1 h, then stimulated with LPS (1 *μ*g/mL) for 24 h. Cell culture medium was collected for ELISA of IL-6 (a) and MCP-1 (b). Cells were pretreated with oridonin at different concentrations for 1 h and then incubated with LPS (1 *μ*g/mL) for 7 or 9 h and total proteins were examined by Western blots with antibody of IL-1*β* (c), or ICAM-1, VCAM-1 (d). GAPDH was used as internal control. Densitometric analyses of bands were quantified and data expressed as fold of control normalized to GAPDH. All Western blot pictures are the representative of at least 3.

To test the anti-inflammatory effects of oridonin in human activated HSCs, we treated LX-2 cells with either LPS alone, or with LPS and varying concentrations of oridonin. The concentrations of oridonin used are 2.5, 5, and 7.5 *μ*M because those concentrations represent the upper dose limit before cell viability in LX-2 cells is affected and the concentrations at which cell viability begins to be affected [18]. In addition, the dose associated with significant levels of apoptosis in LX-2 cells is 6 *μ*M [18]. ELISA was used to determine proinflammatory cytokines secretion. Oridonin significantly decreased LPS-induced IL-6 and MCP-1 secretion in a dose-dependent manner, with significantly decreased levels of IL-6 and MCP-1 for all concentrations of oridonin (P<0.001). (Figures 1(a) and 1(b)). The effect of oridonin on LPS-stimulated IL-1*β* expression was determined by Western blot. As shown in Figure 1(c), LPS increased IL-1*β* protein in a time-dependent fashion and oridonin treatment inhibited LPS-stimulated IL-1*β* expression in a dose-dependent manner. LPS-induced levels of IL-1*β* were significantly increased compared to control at 9 hours (P<0.05). LPS-induced levels of IL-1*β* at 9 hours were significantly decreased with addition of oridonin 7.5 *μ*M (P<0.05).

It has been reported that HSCs are involved in liver inflammation via upregulation of cell surface leukocyte adhesion molecules expression in response to proinflammatory stimulators [22]. To assess the expression of the adhesion molecules ICAM-1 and VCAM-1, total protein was extracted from LPS and oridonin treated LX-2 cells and Western blot was performed. ICAM-1 and VCAM-1 are transmembrane proteins expressed on vascular endothelial cells that serve as a ligand for integrins on immune cells such as lymphocytes, monocytes, eosinophils, and basophils. They serve to promote adhesion and migration of these inflammatory cells from the vasculature into tissues. As shown in Figure 1(d), LPS-induced expression of ICAM-1 and VCAM-1 was significantly increased compared to control (P<0.05 and P<0.001, respectively). Pretreatment with oridonin 7.5 *μ*M significantly decreased LPS-induced expression of ICAM-1 (P<0.05) and pretreatment with oridonin 5.0 *μ*M and 7.5 *μ*M significantly reduced LPS-induced expression of VCAM-1 (P<0.01). In addition, our data demonstrated TLR4 inhibitor TAK-2 44 and NF-*κ*B chemical inhibitors Bortezomib and MG132 blocked LPS-stimulated proinflammatory mediators in LX-2 cells, suggesting the TLR4/NF-*κ*B pathway is involved (data not shown).

### 3.2. Oridonin Blocks LPS-Induced NF-*κ*Bp65 Nuclear Translocation and DNA Binding Activity

**Figure 2:**
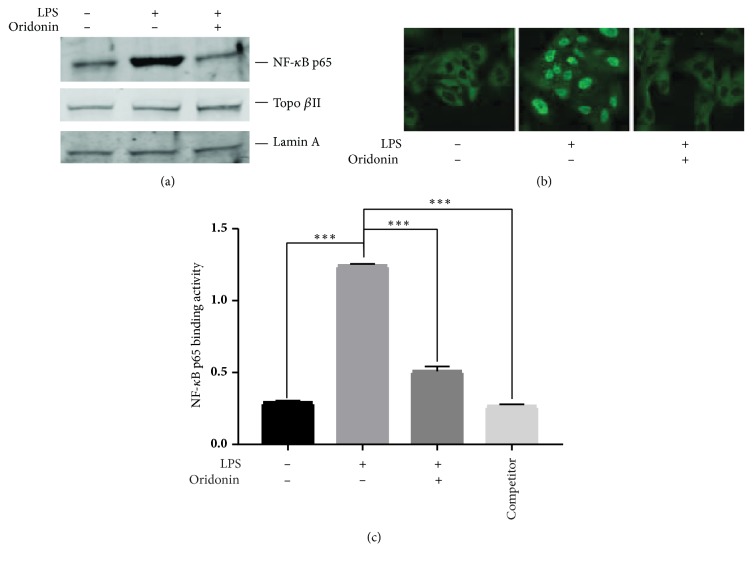
*Oridonin suppresses LPS-induced NF-κB p65 nuclear translocation and DNA binding. *LX-2 cells were pretreated with 7.5 *μ*M of oridonin for 1 h, then stimulated with LPS (1 *μ*g/mL) for 30 min. Nuclear protein was isolated for Western blot assay using NF-*κ*B p65 antibody. Lamin A and Topo *β*II were used as internal control to normalize the nuclear p65 protein (a). The subcellular translocation of NF-*κ*B p65 was imaged by immunofluorescence assay (b). DNA binding activity of NF-*κ*B p65 was analyzed by TransAM p65 ELISA kit with 10 *μ*g of nuclear proteins (c) and 20 pmol of the wild-type NF-*κ*B oligonucleotide as competitor (lane 4). Data are representative of 3 independent experiments.

NF-*κ*B has been demonstrated to be a key component of inflammatory mediator production and plays an important role in liver inflammation. The nuclear translocation of NF-*κ*B subunit p65 is a crucial step in NF-*κ*B pathway activation. Thus, we determined the effects of oridonin on NF-*κ*B p65 translocation from cytoplasm to nucleus with isolated nuclear protein of LX-2 cells. As shown in Figure 2(a), the expression of p65 was markedly increased in the nucleus after 1 *μ*g/mL of LPS treatment for 30 min and reverted to near normal levels with pretreatment with oridonin. This finding was confirmed by immunofluorescence assay, as shown in Figure 2(b). NF-*κ*B p65 sub-cellular localization demonstrated that LPS administration caused NF-*κ*B p65 nuclear translocation and it was prevented by pretreatment with oridonin. To examine whether oridonin affected LPS-induced NF-*κ*B DNA binding activity, we conducted the TransAM NF-*κ*B p65 ELISA assay. As shown in Figure 2(c), LPS-induced NF-*κ*B subunit p65 DNA binding activity was significantly increased compared to control (P<0.001). Pretreatment with oridonin significantly decreased NF-*κ*B p65 DNA binding activity (P<0.001).

### 3.3. Oridonin Affected LPS-Stimulated IKKs/I*κ*B*α*/NF-*κ*B Pathway

**Figure 3:**
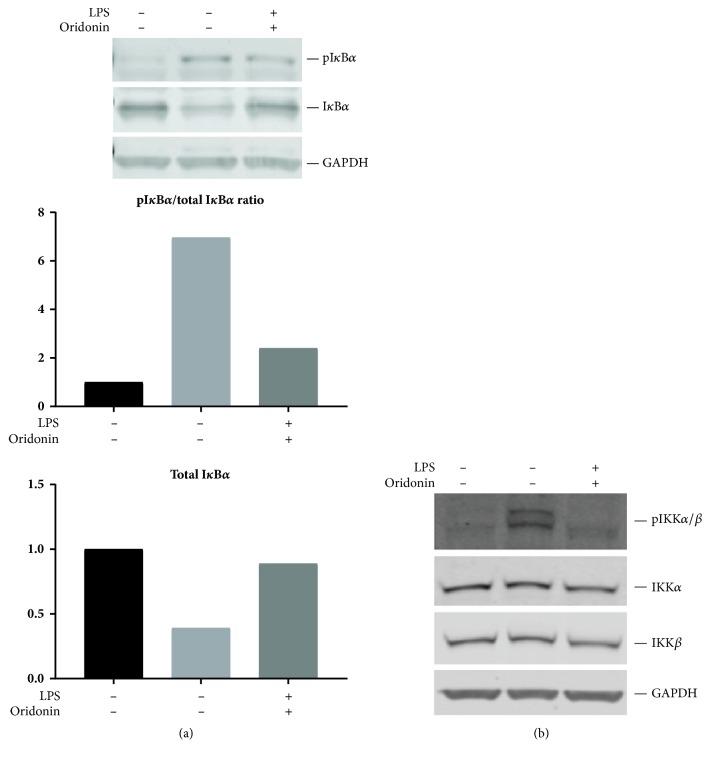
*Oridonin affected LPS-stimulated IKKα/β and IκBα phosphorylation and IκBα degradation. *LX-2 cells were treated with 7.5 *μ*M of oridonin for 1 h followed by LPS (1 *μ*g/mL) for 30 min. Total proteins were examined by western blots using antibody pI*κ*B*α* and I*κ*B*α* (a), or pIKK*α*/*β*, IKK*α* and IKK*β* (b). GAPDh was used as internal control. Densitometric analyses of bands were quantified and data expressed as fold of control normalized to GAPDH. All Western blot pictures are the representative of at least 3 independent experiments.

To further elucidate how NF-*κ*B is affected by oridonin, the protein levels of upstream signaling molecules IKK*α*/*β* and I*κ*B were measured. First, we investigated the effects of oridonin on I*κ*B*α* turnover in response to LPS. As shown in Figure 3(a), LPS treatment for 30 min promoted an increase in the phosphorylation of I*κ*B*α* with a concomitant reduction in cellular I*κ*B*α* protein levels. Pretreatment with oridonin prevented LPS-mediated I*κ*B*α* phosphorylation, while cellular I*κ*B*α* protein level was maintained.

As shown in Figure 3(b), the level of total IKK*α*/*β* was high in all conditions and the basal level of phosphor-IKK*α*/*β*(p-IKK*α*/*β*) was very low. LPS stimulation markedly increased the level of p-IKK*α*/*β*, but oridonin treatment suppressed LPS-stimulated the level of p-IKK*α*/*β*.

### 3.4. Oridonin and PES-CI Synergistically Inhibited LPS-Induced Proinflammatory Cytokines

**Figure 4:**
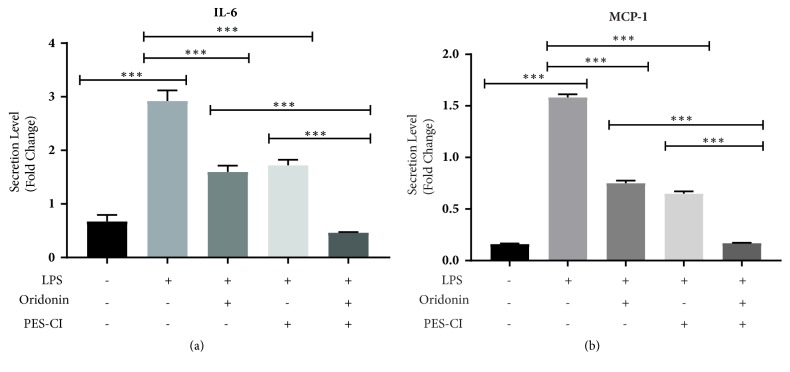
*Oridonin and PES-CI synergistically inhibit LPS-induced proinflammatory cytokines*. LX-2 cells were pretreated with oridonin (2.5 *μ*M), or PES-CI (2.5 *μ*M) or combination of two agents for 1 h, then stimulated with LPS (1 *μ*g/mL) for 24 h. Cell culture medium was collected for ELISA of IL-6 (a) and MCP-1 (b). Data are representative of 3 independent experiments.

It has been established that heat shock protein (Hsp70) is involved in the LPS-induced inflammatory response. A small molecular compound 2-phenylethynesulfonamide (PES), an inhibitor of Hsp70 substrate binding activity, has been reported to attenuate the induction of proinflammatory factors and prevents LPS-induced liver injury [23]. PES-CI is a derivative of PES that demonstrates improved anticancer activity [24, 25]. In the current study, we combined oridonin with PES-CI to treat LX-2 cells at a relatively low concentration (2.5 *μ*M). As shown in Figures 4(a) and 4(b), both oridonin 2.5 *μ*M alone and PES-CI 2.5 *μ*M alone significantly decreased LPS-induced IL-6 and MCP-1 secretion (P<0.001). Combination treatment with both PES-CI 2.5 *μ*M and oridonin 2.5 *μ*M significantly reduced IL-6 and MCP-1 secretion compared to either treatment alone (P<0.001).

### 3.5. The Inhibitory Effects of PES-CI on LPS-Induced NF-*κ*B Signaling Were Enhanced by Oridonin

**Figure 5:**
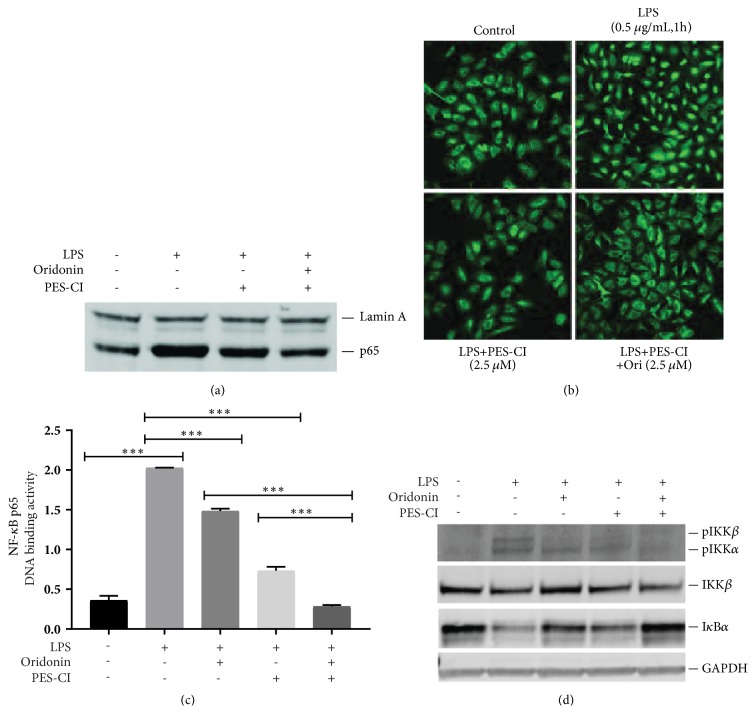
*The inhibitory effects of PES-CI on LPS-induced NF-κB signaling was enhanced by oridonin. *LX-2 cells were pretreated with 2.5 *μ*M of oridonin, PES-CI or combination of two agents for 1 h, then stimulated with LPS (1 *μ*g/mL) for 30 min. Nuclear protein was isolated for Western blot assay using NF-*κ*B p65 antibody. Lamin A was used as internal control to normalize the nuclear p65 protein (a). The subcellular translocation of NF-*κ*B p65 was imaged by immunofluorescence assay (b). DNA binding activity of NF-*κ*B p65 was analyzed by TransAM p65 ELISA kit with 10 *μ*g of nuclear proteins (c). Total proteins were examined by Western blots using antibody pI*κ*B*α*, I*κ*B*α*, or pIKK*α*/*β* (d). Densitometric analyses of bands were quantified and data expressed as fold of control normalized to GAPDH. Data are representative of 3 independent experiments.

To evaluate the effect of combined treatment of oridonin and PES-CI on IKKs/I*κ*B*α*/NF-*κ*B pathway, LX-2 cells were treated with PES-CI at 2.5 *μ*M alone or in combination with oridonin. As shown in Figure 5(a), PES-CI treatment attenuated LPS-induced nuclear NF-*κ*B p65 accumulation; combined treatment markedly enhanced the effects of PES-CI. This observation was confirmed by immunofluorescence staining assay (Figure 5(b)). NF-*κ*B DNA binding activity was determined by TransAM NF-*κ*B p65 DNA binding ELISA. As shown in Figure 5(c), both oridonin (2.5 *μ*M) alone and PES-CI (2.5 *μ*M) alone significantly decreased LPS-induced p65 DNA binding (P<0.001). Combination treatment with both PES-CI (2.5 *μ*M) and oridonin (2.5 *μ*M) led to further significant decreases in p65 DNA binding activity compared to either treatment alone (P<0.001). Interestingly, treatment with either oridonin or PES-CI alone partially recovered LPS-induced I*κ*B*α* degradation. However, total recovery was found with combined treatment (Figure 5(d)). Furthermore, single-agent treatment suppressed LPS-induced pIKK*β* and partially inhibited LPS-induced pIKK*α*, while combined treatment blocked both LPS-induced pIKK*α* and pIKK*β* (Figure 5(d)).

## 4. Discussion

There is increasing evidence showing HSCs not only play an important role in hepatic fibrosis but also contribute to liver inflammation. Activated HSCs promote hepatic inflammation by production of potent chemokines, such as MCP-1 [26]. Endotoxin, such as LPS, was reported to induce IL-8, MCP-1, and MIP-2 in activated HSCs. HSCs also contribute to hepatic inflammation by induction of cell surface of leukocyte adhesion molecules ICAM-1 and VCAM-1 in response to proinflammatory stimulators [22]. Thus, HSCs may participate in hepatic inflammation via enhancement of monocyte and neutrophil transmigration out of the hepatic sinusoid and into the liver parenchyma with endotoxin-involved liver injury. Oridonin, a major ingredient of the traditional Chinese medicinal herb* Rabdosia rubescens,* has anti-inflammatory activity in a variety of cell types. Oridonin has been shown to form a covalent bond with cysteine 279 of the NLRP3 inflammasome, thereby specifically inhibiting NLRP3 inflammasome activation and assembly and suppressing NLRP3-dependent inflammation [27]. NLRP3 is a central inhibitor of innate immunity and inflammation and has been shown to mediate NF-*κ*B activation, making this interaction the likely mechanism of action [28]. Our previous work has focused on the antifibrogenic properties of oridonin and its analogues; however, its anti-inflammatory properties have not yet been fully elucidated in HSCs [18–20]. These previous studies focused on the effect of oridonin on HSC apoptosis, apoptotic markers, and cell-cycle arrest markers without exploring the mechanism of action or hepatic inflammation in response to injury. In the present study, our data revealed that oridonin inhibits LPS-induced proinflammatory mediators MCP-1, IL-6, and IL-1*β* production and adhesion molecules ICAM-1 and VCAM-1 expression in the activated human HSC line LX-2. Simultaneously, we demonstrated that oridonin inhibited LPS-induced NF-*κ*B p65 nuclear translocation and DNA binding activity, while suppressing LPS-stimulated phosphorylation of IKK*α*/*β* and I*κ*B*α* as well as preventing cellular I*κ*B*α* degradation. These results suggest that oridonin not only has potent antifibrogenetic properties but also has effective anti-inflammatory activity in the liver through inhibition of the NF-*κ*B pathway.

Liver injury induced by septic shock is characterized by monocyte and neutrophil infiltration within the parenchymal space. MCP-1, IL-6, and IL-1*β* are potent activators for monocytes and neutrophils. The recruitment of leukocytes to the site of inflammation not only involves chemoattractant factors, such as MCP-1, but also requires adhesion molecules such as ICAM-1 to anchor the leukocytes at the site. Together, these chemokines and adhesion molecules coordinate the migration of leukocytes to the sites of inflammation and injury. Therefore, the inhibitory effects of oridonin for both LPS-induced MCP-1 and ICAM-1 production indicate that oridonin may be an effective compound for treatment of liver injury. A recent paper from Huang et al. confirmed our findings demonstrating that oridonin blocks inflammation via inhibition of NF-*κ*B and MAPK [29]. Their results also demonstrated the blockage of ICAM-1 and VCAM-1. This work was done in human umbilical vein endothelial cells, and our study remains the only one that demonstrates these effects in HSCs. Further studies are necessary to determine the effects of oridonin on other cell types in liver, such as Kupffer cells, hepatocytes, and sinusoidal endothelial cells.

Stimulation with endotoxin leads to activation of NF-*κ*B and production of proinflammatory cytokines. Oridonin is considered to be a natural inhibitor of NF-*κ*B in different cell lines, including Jurkat, RAW264.7 [6], human CD4(+) T cells [30], rat primary microglia [7], and others. Generally, activation of NF-*κ*B involves two important steps: (*a*) phosphorylation and subsequent degradation of I*κ*B*α* by I*κ*B kinases (IKKs), which releases of NF-*κ*B, and (*b*) nuclear translocation of the activated NF-*κ*B subunit p65 and its binding to the target genes' promoter which regulates gene expression. The molecular mechanisms for oridonin's inhibition of NF-*κ*B activity are still not fully understood. It was reported that oridonin interfered with NF-*κ*B DNA binding activity in a number of cell types. Notably, oridonin has an impact on the DNA binding activity and nuclear translocation of NF-*κ*B without affecting I*κ*B-*α* phosphorylation and degradation after TNF*α* treatment in HepG2 cells [31]. In addition, oridonin inhibited NF-*κ*B DNA binding without affecting its nuclear translocation, I*κ*B phosphorylation, and degradation in JurKat cells [6]. In MCF-10A cells, decreases in p65 or p50 forms of NF-*κ*B and I*κ*B were found; however these changes were not seen in MCF-7 cells [32]. All these findings suggest that oridonin regulates NF-*κ*B pathway components in a cell type specific manner. Our data demonstrated that oridonin suppressed LPS-induced NF-*κ*B p65 nuclear translocation and DNA binding, while impairing LPS-induced IKK/I*κ*B*α*/NF-*κ*B signaling in LX-2 cells. The more detailed molecular mechanism of how oridonin regulates the NF-*κ*B pathway should be further evaluated.

The results of the current study showed that a combination treatment with oridonin and PES-CI at relatively low dosage (2.5 *μ*M) synergistically inhibited LPS-induced proinflammatory cytokines, suggesting that combined treatment with oridonin and PES-CI could be a useful therapeutic approach for liver inflammation. Moreover, combined treatment significantly increased the suppression of LPS-induced NF-*κ*B p65 nuclear translocation and DNA binding activity compared to each compound alone. Interestingly, each agent only inhibited LPS-induced phosphorylation of IKK*β*, while combination treatment inhibited LPS-induced phosphorylation of both IKK*α* and IKK*β*.

The inhibitory effect of oridonin on the NF-*κ*B pathway in LX-2 cells suggests that oridonin has a potent anti-inflammatory effect in the liver. Oridonin therefore represents a potential drug candidate for a hepatic anti-inflammatory.

## Data Availability

The data used to support the findings of this study are available from the corresponding author upon request.
